# Automated Acoustic Recogniser Reveals Spatial Variation in Seasonal Calling of the Range‐Restricted Magnificent Broodfrog ( *Pseudophryne covacevichae* )

**DOI:** 10.1002/ece3.74191

**Published:** 2026-08-16

**Authors:** Emily R. Rush, Will Edwards, Lin Schwarzkopf, Slade Allen‐Ankins

**Affiliations:** ^1^ College of Science & Engineering James Cook University Cairns Queensland Australia; ^2^ College of Science & Engineering James Cook University Townsville Queensland Australia

**Keywords:** Australian Wet Tropics, automated acoustic recognition, breeding phenology, Myobatrachidae, passive acoustic monitoring, threatened species

## Abstract

Frog species worldwide are particularly vulnerable to the effects of climate change and are the most endangered vertebrate group. To identify and mitigate impacts, it is necessary to have a method to monitor activity, particularly breeding activity, to detect changes over time. To this end, this study examined the breeding phenology of the poorly known magnificent broodfrog ( *Pseudophryne covacevichae* ) across its disjunct distribution in upland sclerophyll forests of the Wet Tropics of Australia. Using a species‐specific acoustic recogniser, we processed near continuous recordings from November 2022 to November 2023, across ten sites, spanning parts of the species' disjunct ranges, the Atherton Tablelands region and Paluma Range. Recorders sampled nightly for 5 min of every hour and detected ~350,000 advertisement calls using a 95% accuracy threshold. Despite a geographic separation of under 200 km, the timing and duration of calling varied markedly among sites and regions. Atherton Tablelands populations exhibited relatively synchronous, wet season calling, whereas Paluma Range populations had delayed and more prolonged calling periods, extending into the mid to late dry season. Climate window analyses indicated that calling was influenced by antecedent environmental conditions, but the time scale and effect differed between regions. In the Atherton Tablelands, calling intensity was positively associated with high rainfall accumulation over multiple weeks, high minimum temperatures and low humidity over the preceding month. In contrast, in the Paluma Range, calling intensity declined with higher rainfall over the previous 2 weeks and was associated with cooler, but more humid nights in the week prior. The intraspecific variation in calling activity suggests that local hydrological conditions may mediate reproductive timing and duration, with rainfall either facilitating or suppressing calling, depending on the region. Understanding such fine‐scale intraspecific variation in breeding may assist in identifying populations more at risk to the effects of future climate change.

## Graphical abstract

Using acoustic recorders and a species‐specific recogniser we examined the breeding phenology of the magnificent broodfrog *Pseudophryne covacevichae* across ten sites spanning its disjunct distribution in the Australian Wet Tropics over 12 months. The timing and duration of calling differed markedly between regions, with Atherton Tablelands populations calling more synchronously through the wet season and Paluma Range populations exhibiting extended calling into the mid to late dry season. Understanding such intraspecific variation may improve targeted monitoring and identify populations most vulnerable to future climate change.

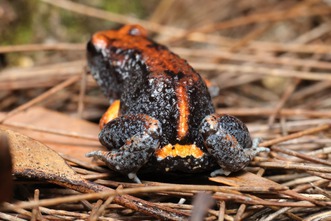

## 1 Introduction

Worldwide, frogs are the most threatened vertebrate group (Luedtke et al. 2023) and they are particularly sensitive to environmental change due to their narrow ecological niches, limited dispersal ability and reliance on moisture (Beebee 1995; Carey and Alexander 2003). A species' breeding phenology can provide insight into their sensitivity and response to different climatic conditions (Parmesan 2006; Green 2017). In amphibians, breeding activity is often closely associated with environmental cues such as rainfall and temperature (Brodie et al. 2025; Chae et al. 2025). Therefore, alterations in the timing, frequency, or intensity of environmental cues used to initiate calling can disrupt or reduce breeding success and affect population viability (Todd et al. 2011; Green 2017). Globally, climatic shifts have already altered amphibian phenology (Walther et al. 2002; Parmesan 2006; Ault et al. 2013), resulting in species initiating or delaying reproduction to times outside of their typical breeding seasons (Beebee 1995; Todd et al. 2011; While and Uller 2014). Phenological responses are complex and species specific, and can reflect reproductive strategy, temperature, moisture availability, habitat type and local climatic variability (Saenz et al. 2006; Ospina et al. 2013; Hoffmann and Mitchell 2022; Amram and Zainudin 2025; Chae et al. 2025). Establishing phenological patterns to understand species' responses to environmental cues are necessary to assess how populations might respond to climate change (Green 2017).

Understanding potential changes in a species' breeding phenology requires methods capable of capturing fine‐scale temporal patterns in behaviour. Passive acoustic monitoring (PAM) provides a non‐invasive, in situ method for monitoring vocal activity in species such as frogs. PAM can provide detailed insight into ecological processes, including the timing and duration of seasonal breeding activity (Heard et al. 2015; Hoffmann and Mitchell 2022; Bolitho et al. 2023; Brodie et al. 2025; Leung et al. 2026), quantifying species richness and diversity (Ramesh et al. 2023; Doohan et al. 2026) and detecting invasive species (Ribeiro et al. 2022; Leung et al. 2025). Species‐specific automated recognisers enable high‐precision detection of target vocalisations across both fine‐scale and landscape‐scale monitoring efforts (e.g., Chae et al. 2025; Leung et al. 2025). This has enabled efficient processing of terabytes of audio data (Kahl et al. 2021; Allen‐Ankins et al. 2025), permitting analyses of species temporal dynamics and responses to environmental change at unprecedented spatial and temporal scales (Roe et al. 2021; Schwarzkopf et al. 2023). By enabling continuous detection of vocal species across large temporal scales, PAM provides an opportunity to track climate‐driven shifts in breeding phenology.

In Australia, approximately 20% of frog species are threatened (Gillespie et al. 2020) and those restricted to the high‐elevation rainforests of the Wet Tropics are considered especially susceptible to climate change (Laurence 2008; Fordham et al. 2016; Geyle et al. 2021). While most research has focused on rainforest taxa in the Wet Tropics, frog species inhabiting the wet and dry sclerophyll forests of the bioregion may be equally vulnerable to emerging stressors altering habitat. The Wet Tropics experiences relatively predictable seasonal weather patterns, with cooler, drier conditions during winter and spring (dry season) and warm, humid weather throughout summer and autumn (wet season). However, these seasonal patterns are projected to be disrupted under future climate change scenarios (Hilbert et al. 2014; McInnes et al. 2015). Over coming decades, ambient temperature across the Wet Tropics bioregion is expected to increase in all seasons. Changes to rainfall patterns are more difficult to model in northern Australia, but there is a predicted tendency toward a reduction in annual rainfall (Hilbert et al. 2014; McInnes et al. 2015).

The upland sclerophyll forests of this region are heterogenous ecosystems shaped by altitude and fire history (Harrington et al. 2000; Stanton et al. 2014) and generally provide less microclimatic buffering than do closed‐canopy rainforests, resulting in greater exposure to elevated temperatures and moisture deficits (Little 2015). Additionally, wet sclerophyll forest is at high risk from increasing frequency and severity of wildfires, driven by climate change, indicated by the 2019/2020 wildfires in which 50% of wet sclerophyll forest and rainforest ecosystems burnt in southeastern Australia (Nolan et al. 2020; Ward et al. 2020). Though sclerophyll forests are well adapted to fire the changing patterns in response to global warming exceeds those experienced in the past, and even frogs adapted to this environment may lack adequate protections (Mahony et al. 2022).

The magnificent broodfrog ( *Pseudophryne covacevichae* ) is a calling, semi‐terrestrial frog species restricted to fragmented pockets of Wet Tropics wet sclerophyll forest (Rush, Edwards, and Hoskin 2025). It is listed as Vulnerable under both the Queensland *Nature Conservation Act 1994* and the Federal *Environment Protection and Biodiversity Act 1999* and Endangered by the International Union for Conservation of Nature Red List (IUCN 2022). Across its range, *P. covacevichae* occupies ephemeral first‐ and second‐order drainage lines between ~700–1100 m on granitic and rhyolitic substrates. Variation in topography across this range gives rise to contrasting habitat types, from drier (herein: xeric) margins of open sclerophyll forest to denser, wetter (herein: mesic) closed sclerophyll forest. These habitats differ in canopy cover and, consequently, in solar radiation, evaporation rates and soil moisture retention. Although the extent of microclimatic variation among *P. covacevichae* populations remains unknown, such environmental heterogeneity is likely to influence local moisture availability and, potentially, the duration of suitable breeding conditions. Reproductive activity is dependent on sustained soil moisture and rainfall (Rush, Hoskin, and Edwards 2025) and even small shifts could shorten a breeding area's hydroperiod, increasing the risk of recruitment failure (e.g., McCaffery et al. 2014). Male *P. covacevichae* broadcast distinct vocalisations to attract females and deter competing males (Groffen et al. 2023; E. R. Rush 2025). Females are rarely encountered (E. Rush 2023) but enter the breeding sites to deposit terrestrial egg clutches within a male's nest. Embryos develop inside the egg to an advanced developmental stage over two‐weeks. Flooding of the nests induces hatching of tadpoles that complete their metamorphosis over some weeks in the adjacent water bodies (Anstis 2017).

Recent genetic analyses indicate that *P. covacevichae* comprises three geographically distinct population clusters across its distribution, corresponding to Atherton Tablelands (central Wet Tropics), Mount Fox and the Paluma Range (southern Wet Tropics) (E. Rush, unpublished data). Long‐term isolation, combined with exposure to different local climatic conditions, raises the possibility of intraspecific variation in seasonal calling behaviour, potentially detectable as population‐specific differences in the timing and intensity of advertisement calling. Their narrow distribution, specialised breeding requirements and reliance on seasonal moisture make *P. covacevichae* particularly susceptible to environmental change. Here, we develop an automated call recogniser capable of detecting *P. covacevichae* calls from audio recordings which we then use to investigate the species advertisement calling phenology over a 12‐month period, to address two primary objectives: (1) investigate intraspecific variation in calling activity; and (2) evaluate the influence of environmental variables on seasonal calling activity. We provide the call recogniser under open access to assist other research and conservation actions for *P. covacevichae* . The results of our study can facilitate future targeted research using PAM, guide the timing of on‐ground survey effort, and develop our understanding of the potential implications of climate change to this threatened species.

## 2 Methods

### 2.1 Study Sites and Acoustic Data Collection

**FIGURE 1:**
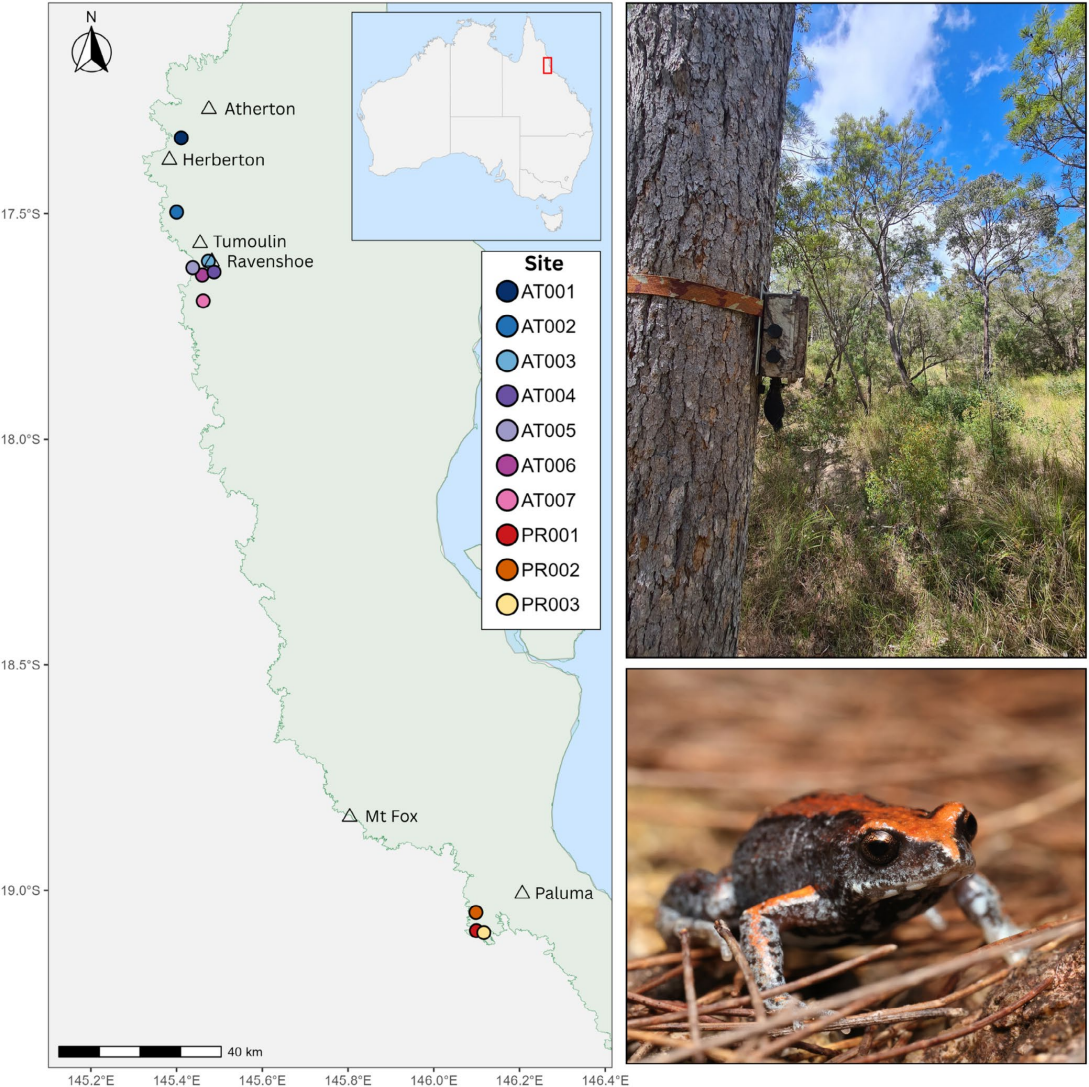
Location of the acoustic monitoring sites in the Wet Tropics region along with nearby towns. The inset map shows the location of the study area within Australia, and the green shading represents the Australian Wet Tropics bioregion. Site names reflect the region: Atherton Tablelands = AT and Paluma Range = PR. A typical set up of the acoustic recorder (top right) and a male magnificent broodfrog ( *Pseudophryne covacevichae* ) (bottom right; photo Patrick Webster).

The study took place in northern Queensland across a geographic expanse of approximately 200 km, spanning montane regions from the southern Atherton Tablelands and southeast to the Paluma Range (Figure 1). Automated recording units (ARUs) were deployed at 13 *P. covacevichae* breeding locations, based on prior records of the species (see Rush, Hoskin, and Edwards 2025). Due to equipment availability, two types of audio recorders: SM4 (Wildlife Acoustics, Maynard, MA, USA) and BAR‐LT (Frontier Labs, QLD, Australia) were used. In the Atherton Tablelands region nine recorders (four SM4 and five BAR‐LT) were deployed, and in the Paluma Range, at the Australian Wildlife Conservancy's Mount Zero‐Taravale Wildlife Sanctuary, four recorders (BAR‐LT) were deployed. A single recording unit was deployed at each site and recorded for approximately one full year (October 2022 until November 2023). ARUs were checked and downloaded every 2–4 months depending on accessibility after rainfall. The study area experiences a tropical upland climate characterised by a pronounced wet season (November–April) and dry season (May–October). The southern Atherton Tablelands and Paluma Range experience similar mean temperatures in summer (29°C–30°C) and winter (12°C–14°C) (Elders Weather 2026a, 2026b), but differ in their average annual rainfall. The Atherton Tablelands experiences approximately 40% less annual rainfall at ~1500 mm in Ravenshoe (Bureau of Meteorology station number 031200) compared to an average of ~2700 mm at Paluma (Bureau of Meteorology station number 032064).

Audio was recorded in WAV format at a sampling rate of 16,000 or 44,100 Hz, exceeding the frequency of *P. covacevichae* vocalisations (E. R. Rush 2025). All ARUs used a single omnidirectional microphone, which was protected by a foam cover encased in malleable metal flywire to reduce invertebrate damage. Units were mounted to trees approximately 1.8 m above ground level and within 2 m of the breeding area (Figure 1). To avoid interference from dawn and dusk bird choruses, ARUs were initially programmed to record for 5 min each hour, every night from 2 h after sunset until 2 h before sunrise. However, as day length increased the original timing schedule caused recordings to miss early calling activity, and schedules were adjusted to record from sunset to sunrise as soon as sites could be accessed.

### 2.2 Development of Call Recogniser

The species' call repertoire includes advertisement, threat and courtship calls (Groffen et al. 2023; E. R. Rush 2025). Due to its role in attracting females, advertisement calls are typically the loudest, most structurally consistent and frequently produced vocalisation in anurans (Gerhardt 1994). Accordingly, we used the species advertisement call to develop a recogniser. To create an initial training dataset, we used a small number of positively identified *P. covacevichae* advertisement calls (*n* = 13) and conducted a similarity search using BirdNET embeddings (Kahl et al. 2021). The embeddings from BirdNET are 1024‐length feature representations of 3‐s audio segments that can be used to find novel sounds outside of the original training data and train custom recognisers. The embeddings of each example call were compared to the embeddings of each 3‐s audio segment in the entire audio dataset using Euclidean distance (see Allen‐Ankins et al. 2025). We then labelled the top 50 closest audio segments (i.e., lowest distance), and 10 randomly sampled audio segments from each of five equally spaced bins across the entire Euclidean distance range per example call.

Using the labelled data from the embeddings search, we trained a generalised‐linear model (glm) using the bayesglm function from the *arm* package, with the BirdNET embeddings as the predictor features and the label (1 = true‐positive, 0 = false‐positive) as the response. The resulting model was then used to predict over the entire audio dataset. To validate model performance, we selected top 10 overall, top 1 per site, 10 random per cut (10 cuts, max 1 per site) and 30 random around 0 logit (to represent audio segments that the current model is most unsure of). This sampling protocol aimed to ensure the model had good performance across all sites.

Using the validated data from above we repeated the model training and validation process for multiple iterations to improve recogniser performance. We also trained an xgboost model to compare performance with the glm on iterations three and four, because an xgboost model can model more complex relationships, potentially improving performance. Both times this model showed a better performance on a held‐out test set (measured as the area‐under the precision‐recall curve PRAUC) than the glm. After four iterations the training dataset had good coverage of the embeddings space of the entire dataset and high performance (Figure S1). The final model was used to predict over the entire dataset with a model probability equivalent to a precision of 0.95 used as a threshold; audio segments exceeding the threshold were labelled as positive detections of *P. covacevichae* .

### 2.3 Environmental Variables

To assess the influence of environmental conditions on *P. covacevichae* calling activity, we compiled a set of variables for each ARU location using gridded data from the SILO climate database, which have a resolution of approximately 5 × 5 km (Queensland Government 2024). Data were extracted using the grid cell that overlaid the ARU location using QGIS (version 3.26.3). Daily measures of rainfall (mm), ambient minimum temperature (°C) and vapour pressure deficit (hPa) were selected based on their demonstrated importance in influencing the vocalising of frog species (Brodie et al. 2025; Chae et al. 2025). Minimum temperature was selected over maximum as it reflects evening temperatures, which is when *P. covacevichae* is most active and when ARUs were scheduled to record. Vapour pressure deficit represents atmospheric moisture, higher values reflect drier air and greater evaporative stress, while lower values indicate more humid conditions. Seasonality was represented by using day of year (DOY) encoded with circular terms, sin(2π DOY/365) and cos(2π DOY/365), derived from each deployment date, enabling cyclic variation in detections across the annual cycle.

### 2.4 Statistical Analysis

The analysis was conducted in two stages: first we characterised site‐level calling phenology. Second, we evaluated the environmental correlates of calling intensity. All analyses were performed using R version 4.5.0 (R Core Team 2025). Audio files were processed using the xgboost recogniser, retaining only those detections above 95% precision. Nightly calling intensity was calculated as the total number of detections per night at each site. Nights with no detections were retained to represent variation in calling activity, including periods of inactivity.

### 2.5 Site‐Level Phenology

To summarise the seasonal timing of *P. covacevichae* calling at each site, we calculated cumulative activity thresholds using nightly call counts. For each site, nightly detections were expressed as a proportion of the total detections and used to construct a cumulative distribution of calling activity. The onset, peak and termination of the breeding season were defined as the 5th, 50th and 95th percentiles of the cumulative distribution, respectively. Because these thresholds were weighted by nightly call intensity, nights with higher activity contributed proportionally more to the cumulative curve, providing an intensity‐weighted estimate of calling activity. The duration of the core calling period for each site was then defined as the number of days between the 5th and 95th percentiles. All calculations were performed separately for each site to capture site‐specific variation. One site was removed from this analysis due to initial recording failure. As only one continuous monitoring period was available per site (i.e., we could only generate a single onset, duration and end date estimate per site), phenological metrics are interpreted descriptively.

### 2.6 Environmental Correlates of Calling

We visually compared calling intensity across the full sampling period and between the Atherton Tablelands and Paluma Range regions to assess broad temporal and regional patterns (Figure S2). To formally test for differences, we fitted a global, negative binomial, generalised linear mixed model (GLMM) using the *glmmTMB* package (Brooks et al. 2017). Calling intensity was modelled as the response variable, with rainfall, region and their interaction included to test for region‐specific rainfall effects. Additional fixed effects were vapour pressure deficit, minimum daily temperature, seasonal timing (sinDOY and cosDOY) and recorder type (either BAR‐LT or SM4). Site was included as a random effect to account for repeated sampling at each location. All continuous predictors were centred and scaled to aid model convergence and interpretation. Multicollinearity among predictors was assessed using model‐based variance inflation factors (VIF) calculated with the *Performance* package (Lüdecke et al. 2021) with VIF > 5 indicating high collinearity. Model fit was evaluated using Akaike's Information Criterion (AIC) and parameter estimates interpreted on the log scale. Model diagnostics, including checks for dispersion, zero inflation and residual structure, were conducted using the *DHARMa* package (Hartig and Hartig 2017). This global model revealed strong effects of environmental and seasonal variables on calling activity of *P. covacevichae* , and a strong interaction between region and rainfall. Given the pronounced regional interaction, all subsequent analyses were conducted separately for the Atherton Tablelands and Paluma Range to accurately capture the measured environmental effects on *P. covacevichae* calling.

Due to the potential for environmental conditions to have lagged effects on calling (Rush, Hoskin, and Edwards 2025) we investigated short‐term weather influences on calling intensity, using a sliding window analysis with the *climwin* package (Bailey and van de Pol 2016; van de Pol et al. 2016). This approach identifies the time window over which a climatic variable best explains variation in a biological response. Climate window analyses were conducted separately for the Atherton Tablelands and Paluma Range regions, and for each climate variable (daily rainfall, vapour pressure deficit and minimum temperature). For each variable, all possible time windows within the 30 days preceding each biological observation, using a relative time approach and a daily resolution, were evaluated. Within each candidate window, the climatic variable was summarised using the mean value. Following initial tests, baseline models in the Atherton Tablelands were fitted as a ‘glmr.nb’ and in the Paluma Range as a ‘glmr’ using a negative binomial error distribution. In all baseline models, calling intensity was the response variable and the climate window metric was a fixed effect, with site as a random intercept. Model support was assessed using ΔAICc relative to a baseline model. To assess the robustness of the climate windows and account for overfitting, randomisation tests were conducted using the *randwin* function with 100 repeats per variable. Climate windows were considered well supported if their observed ΔAICc exceeded those obtained from the randomised datasets. The *p* value was calculated with the metric set to ‘AIC’ (Bailey and van de Pol 2016).

For each region, the best‐supported climate windows identified in the sliding window analyses were re‐incorporated into negative binomial GLMMs to explore the response of the climate windows to *P. covacevichae* calling activity. These models retained seasonal covariates (sinDOY and cosDOY) to account for underlying cyclic patterns in calling activity independent of short‐term climatic effects and included site as a random intercept. For the Atherton Tablelands model we also included recorder type as a fixed effect. All continuous predictors were centred and scaled to facilitate model convergence and the effects are presented as log‐link, interpreted as proportional changes in expected calling intensity. Model diagnostics were again evaluated using the *DHARMa* package and multicollinearity was assessed using VIF. To visualise the effects of climatic and seasonal predictors on calling intensity, predicted relationships were generated from the final fitted models for each region. Predicted calling intensity and corresponding 95% confidence intervals were plotted.

## 3 Results

No *P. covacevichae* were detected by the recogniser at three sites (two northern and one southern) and the data from those ARUs were subsequently removed, leaving 10 sites for the analyses (Figure 1). Some equipment malfunctions meant that audio was not recorded every day of the deployment period. Visual inspection revealed the most consistent window of recording, when all sites had functional recorders, was between the 6th November 2022 and 6th November 2023 (Figure S3), though this differed slightly in some instances (see Table 1).

**TABLE 1:** Site specific phenology thresholds for *Pseudophryne covacevichae* .

Site	Onset	Peak	End	Duration of core breeding season	Total calls	Recorder start date	Recorder end date	Total rainfall (mm)
AT001	19/12/2022	10/01/2023	20/02/2023	63	15,996	06/11/2022	12/09/2023	1479
AT002	06/12/2022	13/01/2023	04/04/2023	119	17,776	20/11/2022	28/10/2023	1583
AT004	04/12/2022	26/01/2023	23/03/2023	109	51,293	6/11/2022	06/11/2023	1770
AT005	28/11/2022	21/12/2022	15/02/2023	79	2829	6/11/2022	06/11/2023	1627
AT006	30/11/2022	10/12/2022	08/02/2023	70	1408	6/11/2022	06/11/2023	1552
AT007	26/11/2022	22/01/2023	19/04/2023	144	63,828	6/11/2022	6/11/2023	1525
PR001	18/04/2023	06/06/2023	29/07/2023	102	66,265	6/11/2022	6/11/2023	1064
PR002	09/05/2023	08/07/2023	06/10/2023	150	53,974	6/11/2022	6/11/2023	1315
PR003	12/11/2022	27/04/2023	12/07/2023	242	79,022	6/11/2022	6/11/2023	1064

*Note:* Onset = first day at which 5% cumulative calls were reached, peak = 50% cumulative activity and end = 95% cumulative activity. Duration of the core breeding season is the number of sampling days between the 5% and 95% cumulative activity thresholds. Total calls represent the sum of all detections recorded for that site. Start and end dates for the recording periods at each site are shown, as well as the total rainfall from SILO data.

### 3.1 Call Recogniser

**FIGURE 2:**
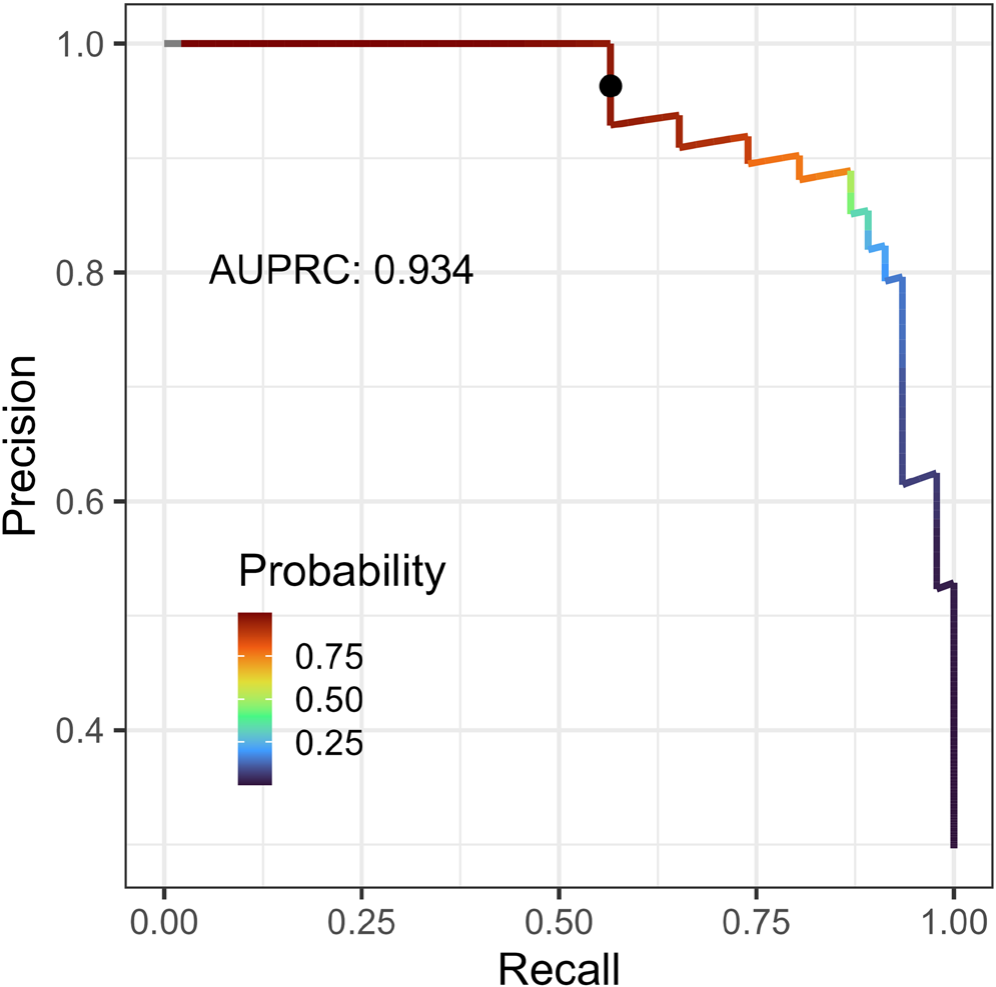
Precision‐recall curve of final xgboost model showing model performance on a held‐out validation set (*n* = 155) across all probability thresholds. The final model had an Area Under the Precision‐Recall Curve (AUPRC) of 0.934. The black dot represents the probability threshold (0.957) used to filter the final predictions.

After four training iterations, the final xgboost model had an AUPRC of 0.934 on the final held‐out test dataset (Figure 2). The final training dataset for the *P. covacevichae* advertisement call recogniser comprised 890 positive labels and 821 negative labels. We provide both the final glm and the xgboost model for use. The glm may be safer to use on new data than the xgboost model, especially if the embeddings of a new dataset are very different to that of the dataset used to train our model. We also provide a final model trained using the BirdNET GUI in .tflite format for use within that application and in a format for use with Raven Pro (Cornell Lab of Ornithology). This version is intended to be convenient and easy‐to‐use for those unfamiliar with the R statistical environment.

### 3.2 Site‐Level Phenology

**FIGURE 3:**
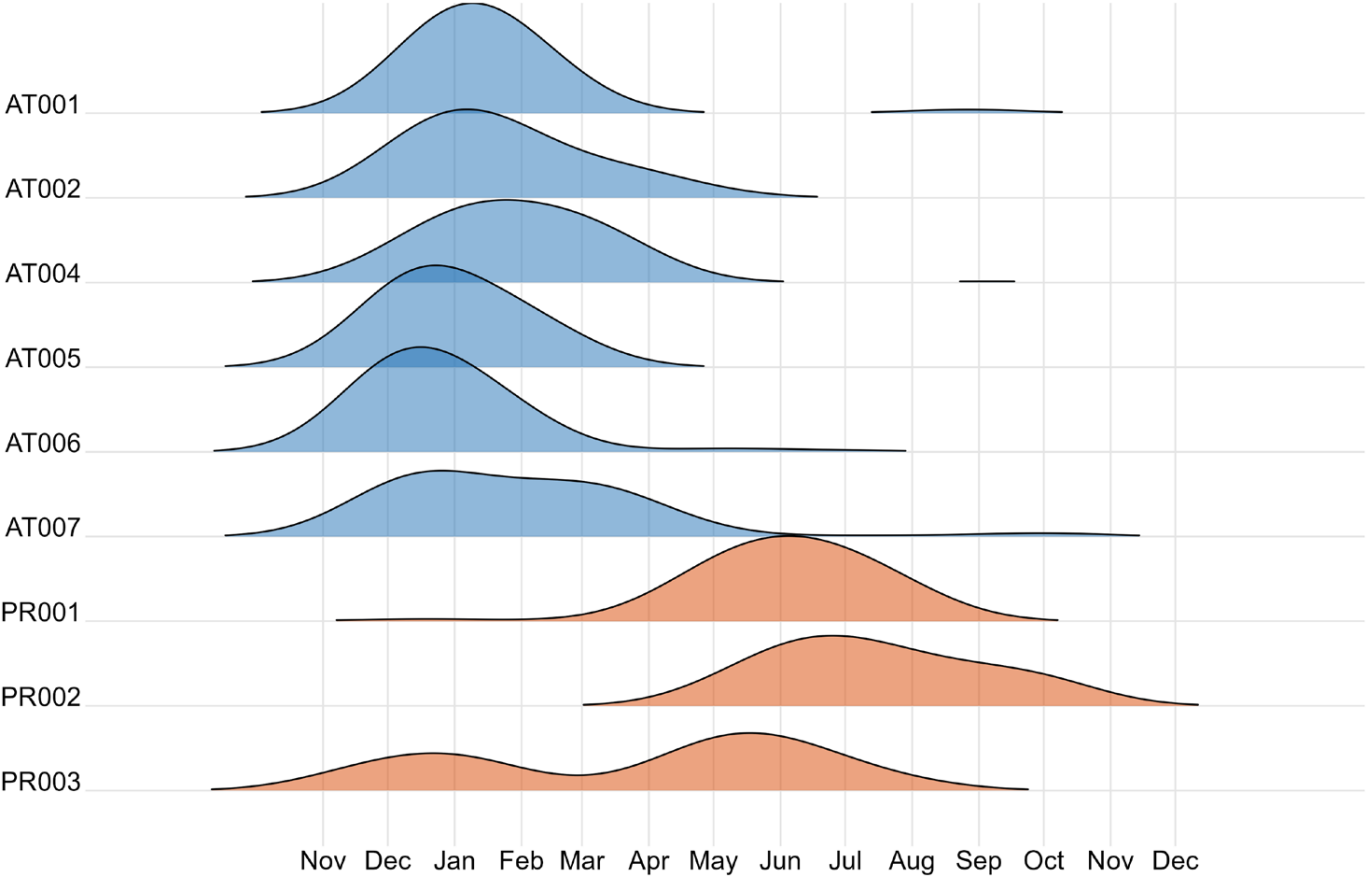
Ridgeline density plot of the monthly distribution of calling activity of *Pseudophryne covacevichae* across the Atherton Tablelands (blue) and Paluma Range (orange) sites. Each ridge represents a site, arranged from north to south. Ridge heights show kernel density estimates of calling activity weighted by nightly call counts, with higher peaks indicating more frequent calling.

There was substantial variation in the number of detections among sites (1408–79,022; Table 1). While both regions had highly active sites, the highest total call counts were recorded at the three Paluma Range sites and one Atherton Tablelands site (AT007). Total SILO rainfall over the study period was higher in the Atherton Tablelands compared to the Paluma Range sites and the timing and duration of *P. covacevichae* calling activity differed between regions (Figure 3; Table 1). In the Atherton Tablelands, calling was strongly seasonal with peak activity occurring during the wet season between December and March when monthly rainfall was higher (Table S5). In contrast, Paluma Range sites exhibited later seasonal peaks, predominantly during the early to mid‐dry season (May–July) (Figure 3) when rainfall had subsided (Table S5). One Paluma Range site (PR003) had calling sustained across much of the sampling period and a bimodal pattern characterised by peaks in both December and May.

Cumulative activity thresholds supported these regional differences. Onset and peak of core breeding activity (5% and 50% cumulative calls) occurred at a similar time in the seven Atherton Tablelands sites and at one Paluma Range site (Nov–Feb; Table 1), while onset and peak calling at the two other Paluma Range sites occurred months later between March and July (Table 1). In the Atherton Tablelands, calling intensity was greatly reduced (95% cumulative calls) by April but continued as late as October in the Paluma Range. The duration of core breeding (5%–95% cumulative activity) ranged from 63–144 days at Atherton Tablelands sites and from 102 to 242 days at Paluma Range sites, indicating some comparable or longer breeding events in the southern region. The one exception was PR003, which exhibited two breeding peaks and an extended breeding duration compared to all other sites (Figure 3; Table 1).

### 3.3 Environmental Correlates of Calling

In the global model, rainfall had a significant positive effect on calling activity in the Atherton Tablelands (*β* = 0.150 ± 0.025, *p* < 0.001), but this relationship differed significantly between regions (*z* = −9.39, *p* < 0.001) with a negative effect of rainfall in the south (*β* = −0.384 ± 0.057, *p* < 0.001). The effect size of recorder type was small and not significant (*β* = 0.039 ± 0.500, *p* = 0.938), corresponding to an approximate 4% increase in detections for SM4 recorders relative to BAR‐LT. However, the large standard error indicates substantial uncertainty around this estimate. With limited replication (three SM4 vs seven BAR‐LT), statistical power to detect differences was low. Variation among sites, modelled as a random effect, accounted for substantial variability in detections (*σ* ^2^ = 0.418), implying that differences among sites were greater than the effect of recorder type.

In the Atherton Tablelands, the sliding window analysis revealed that rainfall had the strongest relationship to calling, with mean rainfall over preceding 25–0 days identified as the optimal weather window, indicating that calling activity increased following sustained wet conditions (Table 2). Minimum temperature also exhibited a positive association with calling intensity, with the best‐supported window spanning 29–0 days, suggesting that warmer temperatures over several weeks promoted increased calling. Vapour pressure deficit had a shorter window of influence, with the strongest support for the 30–13 day period, indicating increased calling intensity under relatively drier atmospheric conditions (i.e., when rainfall is higher, humidity was reduced) during the 2 weeks preceding calling. These patterns suggest that in the northern sites, calling is influenced by overlapping antecedent climatic conditions that build over several weeks rather than daily weather (Table 2; Figure S4).

**TABLE 2:** Results of the climate window analyses for climate predictors influencing *Pseudophryne covacevichae* calling intensity.

Predictor	Window opens	Window closes	∆AICc	*β* (SE)	*Z*	*p*
Atherton tablelands (*n* = 7)						
Rain (mean)	25	0	−354.380	6.263 (0.384)	16.29	< 0.001
minTemp (mean)	29	0	−441.270	2.375 (0.102)	23.28	< 0.001
Vapour pressure deficit (mean)	30	13	−106.376	1.478 (0.161)	9.142	< 0.001
Paluma range (*n* = 3)						
Rain (mean)	30	11	−698.913	−2.556 (0.083)	−30.75	< 0.001
minTemp (mean)	8	1	−553.981	−1.023 (0.048)	−21.82	< 0.001
Vapour pressure deficit (mean)	3	0	−692.282	−1.244 (0.043)	−28.72	< 0.001

*Note:* Best supported opening and closing windows (in days prior to detections) and associated ∆AICc, model coefficients (*β* ± SE) and test statistics (*Z*, *p*).

In the Paluma Range, sliding window analyses identified negative relationships between calling intensity and all three environmental variables, with each predictor exerting its strongest effect over a shorter period (Table 2; Figure S5). Cumulative rainfall over a 30–11 day window showed the largest effect, followed by minimum temperature over the 8–1 day window. Vapour pressure deficit had a short optimal window, with the strongest support for the 3–0 day period, when atmospheric moisture was higher (i.e., lower vapour pressure deficit). These results indicate that, unlike the Atherton Tablelands sites, calling was most closely associated with shorter antecedent periods and was suppressed when rainfall and minimum temperature were higher and vapour pressure deficit was lower, over those windows.

In all instances, the randomisation tests confirmed that the climate relationships identified via climwin were unlikely to arise by chance. Observed effect sizes consistently exceeded those from the randomised datasets, with large reductions in ΔAICc relative to null models (*p* < 0.001 for all variables; Table S1, Figures S4 and S5).

**FIGURE 4:**
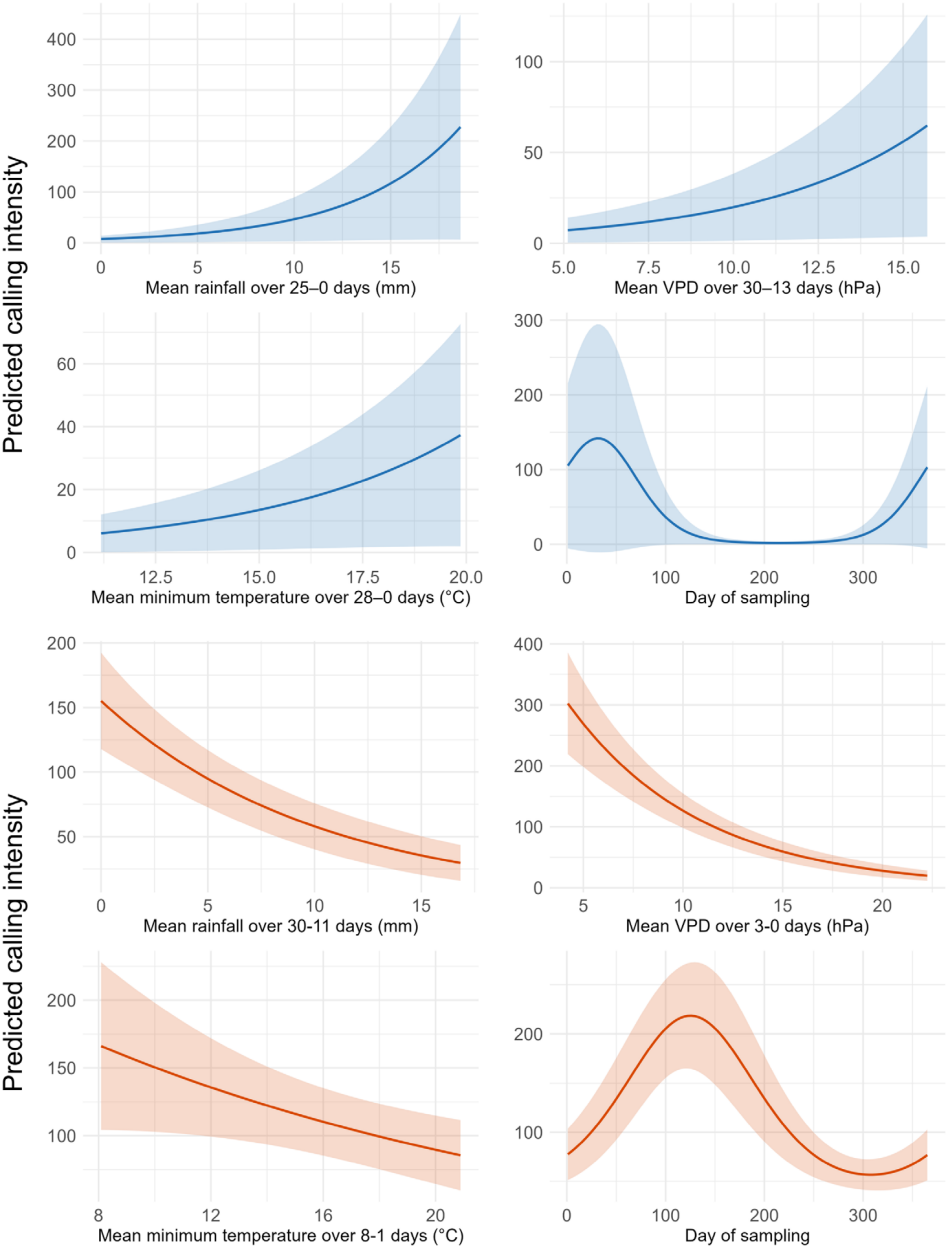
Predicted effects of rainfall, vapour pressure deficit (VPD), minimum temperature and seasonal timing (Day of sampling) on *Pseudophryne covacevichae* calling intensity at Atherton Tablelands sites (blue) and Paluma Range sites (orange). The seasonal timing panel in the Atherton Tablelands is shown for comparison only and was fitted in the model that included DOY terms (not the model that the plotted environmental variables are based on). Shaded areas represent 95% confidence intervals and the sampling period ranges from 06/11/2022 (Day 1) to 06/11/2023 (Day 366).

In the Atherton Tablelands, variance inflation factors indicated high collinearity (VIF > 5) among predictors when seasonal terms (DOY) were included in a GLMM. Removing DOY terms reduced VIF values to < 5. In the reduced model, which included the ‘best’ model result from *climwin* analysis, rainfall, minimum temperature and vapour pressure deficit were all significant predictors of calling intensity (Figure 4; Table S2). Calling intensity increased with rainfall over the preceding 25 days (*β* = 2.357 ± 0.146, *p* < 0.001), indicating substantially higher expected calling intensity under wetter prior conditions. Minimum temperature (*β* = 0.602 ± 0.090, *p* < 0.001) and vapour pressure deficit (*β* = 0.822 ± 0.068, *p* < 0.001) also showed strong positive associations with calling intensity. Recorder type was more evenly represented when Paluma Range sites were excluded (three SM4 and four BAR‐LT); however, the effect of recorder type remained non‐significant (*β* = 0.474 ± 0.663, *p* = 0.507). The large standard error indicates substantial uncertainty around this estimate, suggesting that any differences between the two recorder types were not reliably detected. Site‐level variation was large (*σ* ^2^ = 0.868) and model diagnostics suggested no issues with dispersion or zero inflation.

The same modelling approach applied to the Paluma Range sites revealed no collinearity when seasonal terms (DOY) were included in the model. In contrast to the Atherton Tablelands, all three environmental variables were negatively associated with calling intensity (Figure 4; Table S2). Calling intensity declined with increasing rainfall in the 2 weeks before an observation (*β* = −0.857 ± 0.131, *p* < 0.001), indicating reduced calling activity following wetter conditions. Vapour pressure deficit also showed a strong negative effect (*β* = −0.656 ± 0.064, *p* < 0.001), while minimum temperature exhibited a weaker but still significant negative relationship with calling intensity (*β* = −0.184 ± 0.071, *p* = 0.009). Seasonal timing was an important predictor of calling intensity (sinDOY: *β* = 0.562 ± 0.068, *p* < 0.001; cosDOY: *β* = −0.372 ± 0.121, *p* < 0.002), indicating a pronounced annual cycle independent of short‐term climate variation. Site‐level variation was lower in the Paluma Range (*σ* ^2^ = 0.028), indicating more consistent calling intensity among sites, relative to the Atherton Tablelands. Model diagnostics showed no evidence of zero inflation but some under‐dispersion was detected relative to the negative binomial variance assumption (dispersion = 0.49, *p* < 0.001) (Hartig and Hartig 2017). A bootstrapped outlier test confirmed that there were no extreme outliers in the model (*p* = 0.98).

## 4 Discussion

Using a small number of example calls, we were able to leverage a pre‐trained deep learning recogniser (i.e., BirdNET) to efficiently develop an accurate call recogniser and obtain calling patterns of a threatened species at a fine temporal scale. The analysis of calling patterns and influence of environmental variables was based on over 350,000 *P. covacevichae* calls, automatically detected in approximately 3000 h of recorded audio over 12 months. Across the annual cycle and within a small geographic expanse, populations of *P. covacevichae* exhibited pronounced regional differences in the timing and duration of calling activity. Atherton Tablelands sites initiated and completed calling earlier, with calling aligned to the wet season, whereas Paluma Range sites varied but generally called more in the early to mid‐dry season, and at one site, there was sustained calling across much of the sampling year. Analyses using cumulative windows of rainfall, minimum temperature and vapour pressure deficit revealed the effects of these variables act in opposing directions, indicating that males responded differently to combinations of moisture and atmospheric conditions between the two regions. Collectively, these results highlight that *P. covacevichae* may not follow a uniform phenological pattern across its small range. We propose that the regional differences could be explained by local hydrology, forest structure and moisture retention capacity between sites rather than rainfall alone.

### 4.1 Site‐Level Phenology

Over the study period the SILO data suggested that sites in the Atherton Tablelands received higher total rainfall than those in the Paluma Range (Table 1; Table S5). However, higher rainfall did not necessarily translate to greater calling activity or longer breeding durations. For example, some Atherton Tablelands sites with relatively high annual rainfall recorded some of the lowest call totals and shorter breeding durations, while some lower rainfall sites had the highest calling activity and longest breeding duration (Table 1). Atherton Tablelands sites exhibited earlier onset and completion of core breeding activity compared to the Paluma Range sites (with the exception of PR003), which showed delayed onset and extended calling activity later into the year. The variation indicates that reproductive responses are not directly linked to broad climate alone and instead may be structured by fine‐scale landscape features that influence a site's microclimate.

In terrestrial and semi‐terrestrial breeding anurans, including *Pseudophryne* species, soil moisture governs both adult hydration and the viability of terrestrial egg clutches (Mitchell 2002; O'Brien et al. 2020). Consequently, precipitation and its associated effects at a site (e.g., soil moisture) likely contribute to the observed differences we document in *P. covacevichae* . Presumably, the capacity of a site's hydrology and soil water potential mediates the window over which successful reproduction is possible, as documented in another myobatrachid species the white‐bellied frog, *Anstisia alba* (Hoffmann and Mitchell 2022).

Forest structure influences evaporation and distribution of sunlight, wind and other atmospheric factors, which act alongside topographic features such as slope, aspect, hydrology and water availability to shape microclimate (Zellweger et al. 2019; De Frenne et al. 2021). Canopy cover, understorey density and leaf litter can alter evaporation rates and hence, soil moisture persistence (Turton and Duff 1992; Little 2015). As a result, sites receiving similar rainfall can differ substantially in moisture availability (Little 2015). In this context, the xeric sites we refer to do not necessarily receive less rainfall and have higher temperatures but rather may have less capacity to retain moisture due to higher solar radiation and evaporation rates (e.g., Figure S6). Conversely, mesic sites may be able to maintain soil moisture and standing water for longer periods regardless of the amount of rainfall they receive (see Little 2015). This is reflected in the patterns we observed where longer breeding durations and higher call counts occurred at sites characterised by mesic sclerophyll forest (Table S3). These sites may experience less variability in soil moisture relative to more exposed sites, reflecting the buffering effects of canopy cover on evaporation and soil moisture (Suggitt et al. 2018; De Frenne et al. 2021), though this was not directly measured in our study.

Acoustic detections can be a reflection of population density (Marques et al. 2013; Pérez‐Granados and Traba 2021; Stevenson et al. 2021) and the detection totals of *P. covacevichae* broadly mirrored anecdotal field observations of population size (E. Rush, pers. observation), though these observations were not systematically collected and would require validation for acoustic abundance estimates. As an example, PR003, a seepage site, recorded the highest acoustic call totals and consistently high male densities in the field, whereas Millstream National Park (AT006) and Ravenshoe State Forest (AT005) produced substantially fewer call detections (1408 and 2829, respectively), consistent with lower field observed abundance. These observations suggest that forest structure and associated microclimate may influence the duration and intensity of *P. covacevichae* reproductive activity and, by extension, population density. At xeric sites, suitable moisture conditions may be reached for shorter periods, compressing breeding duration into smaller windows and limiting opportunities for recruitment. In contrast, where prolonged reproductive windows can occur, there may also be an increase in recruitment potential. Similar relationships between hydroperiod length, breeding opportunity and recruitment have been observed in other amphibians (Baldwin et al. 2006; Karraker and Gibbs 2009; McCaffery et al. 2014). For example, the Columbia spotted frog ( *Rana luteiventris* ), which also occurs in heterogeneous habitats and exhibits high site fidelity, shows higher recruitment in waterbodies that have longer hydroperiods (McCaffery et al. 2014). Overall, our results suggest that calling activity is structured primarily by the persistence of moisture, with site‐specific hydrology likely defining the timing and duration of reproduction.

### 4.2 Environmental Correlates of Calling

In the Atherton Tablelands, calling increased following several weeks of warm, wet conditions. The longer prior climate windows here suggest that calling is promoted by the gradual establishment of favourable hydrological conditions rather than isolated rainfall events. This result is consistent with previous research, where *P. covacevichae* detectability during on‐ground surveys increased following sustained increased soil moisture and higher accumulated prior rainfall (Rush, Hoskin, and Edwards 2025). In contrast, all three climatic windows in the Paluma Range were negatively associated with calling and their influence operated over shorter time frames. Here, calling increased following periods of reduced rainfall in the preceding 2 weeks and on nights that were cooler and atmospheric moisture was higher (i.e., low vapour pressure deficit). Early wet season detections did occur at Paluma Range sites, but calling intensity was substantially lower than later in the sampling period (Figure S2). The negative rainfall relationship observed in the Paluma Range suggests that once moisture exceeds a functional threshold, additional rainfall may suppress calling rather than facilitate it.

The converse rainfall relationship observed between the Paluma Range and the Atherton Tablelands suggests that climatic conditions, or local hydrology, or both, differ sufficiently between these locations to provide contrasting timing for peak calling and hence, reproduction. In *Pseudophryne* species, egg clutches are positioned in an area where water flows over but does not remain standing after heavy rainfall (Woodruff 1977; Thumm and Mahony 2002). This is because during the terrestrial phase, *Pseudophryne* eggs are extremely susceptible to both desiccation (Woodruff 1976; Mitchell 2001; Eads et al. 2012) and incorrect timing of flooding and hatching (Geiser and Seymour 1989). If inundated too early, or too late, there is a risk that subsequent standing water will not be available to support tadpole metamorphosis (Geiser and Seymour 1989). The timing of egg laying is therefore crucial in reducing the risk of clutch mortality. Reproductive timing may therefore be under strong selective pressure to align with local moisture conditions. The species may either adjust breeding behaviour plastically in response to available moisture, or be locally adapted to their respective environment, resulting in different phenological responses.

The contrasting patterns observed between the Atherton Tablelands and Paluma Range suggest that reproductive activity may have been influenced less by absolute rainfall and more by how hydrology influences moisture retention within local environments. Although the Paluma region typically receives substantially higher annual rainfall than the Atherton Tablelands (Table S4) (Bureau of Meteorology 2026a, 2026b, 2026c), interpolated SILO data indicated that Atherton Tablelands sites experienced comparable or greater monthly rainfall over the study period (Table S5). In the Atherton Tablelands, calling intensity declined markedly after May, even at more mesic sites, suggesting that in this area wet season rainfall didn't support calling (and hence reproductive activity) throughout the dry season. In contrast, the delayed and prolonged calling observed in the Paluma Range sites may reflect greater climatic buffering and hydrological capacity, for example, through topographic position, soil composition, or canopy structure that allowed favourable moisture conditions (i.e., dry season soil moisture) to be retained longer into the study year, despite potentially lower rainfall input, as evidenced by the SILO dataset.

The negative relationship between rainfall and calling intensity in the Paluma Range suggested that high rainfall reduced reproductive output. If these sites do have a substantially wetter microclimate there is potential that the shallow breeding areas reach saturation early in the wet season, potentially increasing the likelihood of nest inundation during a period when embryonic development is still underway (Geiser and Seymour 1989). Egg development in *P. covacevichae* has only been documented for a population in the Atherton Tablelands and is relatively short (~11 days) (Anstis 2017). If the duration of terrestrial development differs in the Paluma Range, it could increase vulnerability to premature inundation. Delaying reproduction until later in the season, when rainfall subsided, may therefore reduce the risk of nest failure. Soil moisture, rather than direct rainfall, could therefore be a key ecological constraint for the species, which is consistent with its distribution being coupled to dry‐season rainfall (Rush, Edwards, and Hoskin 2025). Investigating fine‐scale habitat, including canopy cover, slope and dry season soil moisture, may aid in determining how forest structure, hydrology and microclimate influences breeding activity and persistence across the sites and regions (e.g., Hoffmann and Mitchell 2022).

The extent to which regional differences reflect local adaptation vs phenotypic plasticity is unclear. Strong genetic structuring has been documented between Atherton Tablelands, Mount Fox and Paluma Range populations (E. Rush, unpublished data) suggesting long‐term isolation, which could drive adaptation to local conditions and hence the regional differences in reproductive timing we observed. If differences in reproductive timing are shaped by local adaptation, then populations may have difficulty adjusting breeding responses under changing climatic conditions (Phillimore et al. 2010; Cayuela et al. 2019). Under this scenario, populations occupying xeric habitats where observed breeding windows were shorter and recruitment may potentially be lower are likely to be disproportionately vulnerable to shifts in rainfall and increased climatic variability, predicted in the Wet Tropics (Hilbert et al. 2014; McInnes et al. 2015). In contrast, if breeding in *P. covacevichae* is primarily facultative, individuals may adjust reproductive timing, or skip reproduction entirely, in response to environmental stochasticity. Such plasticity has been widely documented in amphibians (Walls et al. 2013; Ficetola and Maiorano 2016; Cayuela et al. 2019) and would be advantageous for *P. covacevichae* , allowing them to synchronise breeding with favourable environmental conditions within the heterogenous habitats they occupy. Resolving these mechanisms would require experimental approaches, such as common garden experiments and reciprocal‐transplant studies (Blanquart et al. 2013; de Villemereuil et al. 2016), or continuous long‐term acoustic monitoring to investigate the response to varying annual rainfall.

A caveat of this study is that heavy rainfall may reduce the ability of the recogniser to detect calls (Ferroudj et al. 2014; Willacy et al. 2015; Zhang et al. 2024). Anecdotal field observations during the same sampling period indicated that male *P. covacevichae* reduced calling during intense rainfall events (E. Rush, pers. observation), potentially because signal transmission to females is compromised when there is extensive environmental noise. Behavioural suppression of calling during heavy rainfall is documented in some anuran species (Ospina et al. 2013; Willacy et al. 2015), though in others it encourages calling (Saenz et al. 2006; Brodie et al. 2025). A further consideration is that we used ambient temperature as a proxy for thermal conditions experienced by calling males. Substrate or microhabitat temperatures at calling sites may better reflect the temperatures experienced by individuals and could improve understanding of the relationship between temperature and calling activity (Scheffers et al. 2014; Bolitho et al. 2023).

Our findings are representative of a single year and annual variation in rainfall is likely to influence both the timing and magnitude of calling responses in both the Atherton Tablelands and Paluma Range. Long‐term acoustic monitoring across multiple years, including at Mount Fox, will be necessary to determine whether the climate–calling relationships identified here remain consistent under different rainfall regimes. Expanding monitoring to additional Paluma Range sites in both xeric and mesic habitats would further clarify how microclimate and hydrology shape regional breeding patterns in this area. The automated recogniser provides a practical tool to support monitoring. Until then, our findings suggest the timing of field surveys requires flexibility especially if the species response to environmental variables is facultative; regardless, they should be targeted toward periods when soil moisture is high.

Using 12 months of acoustic data, we establish a baseline for *P. covacevichae* breeding activity, providing previously undocumented detail to inform future research. Our results demonstrate that breeding phenology varied across the range of *P. covacevichae* , despite populations occupying a broadly similar climatic region. This regional variation suggests that differences in local hydrology and forest structure may influence the duration of breeding activity and highlights that populations may respond differently to future environmental change. If rainfall becomes less reliable under future climate change, reduced moisture availability could constrain breeding opportunities and limit recruitment in already small populations. These findings suggest that broad‐scale climatic variables alone may be insufficient to assess population vulnerability and that local habitat characteristics and associated hydrological conditions should also be considered when evaluating the potential impacts of climate change on different populations. Long‐term passive acoustic monitoring provides a valuable tool for detecting changes in breeding activity that may provide early warning of population decline.

## Author Contributions

**Emily R. Rush:** conceptualization (lead), data curation (lead), formal analysis (lead), funding acquisition (lead), investigation (lead), methodology (lead), project administration (lead), writing – review and editing (lead). **Will Edwards:** conceptualization (equal), formal analysis (supporting), funding acquisition (supporting), investigation (supporting), methodology (equal), project administration (equal), supervision (lead), writing – review and editing (equal). **Lin Schwarzkopf:** investigation (supporting), methodology (supporting), writing – review and editing (supporting). **Slade Allen‐Ankins:** conceptualization (supporting), data curation (equal), formal analysis (supporting), investigation (supporting), methodology (equal), software (lead), supervision (supporting), writing – review and editing (supporting).

## Funding

This research was funded by a Nature Refuge Landholder Grant (NRLG) accessed through the Australian Wildlife Conservancy, a Holsworth Wildlife Research Endowment Grant (Ecological Society of Australia), a donation from the Magnificent Broodfrog Working Group, and a Skyrail Rainforest Foundation grant.

## Ethics Statement

This research was conducted under James Cook University animal ethics approval A2839 and Queensland Government scientific research permits P‐PTUKI‐100274044, P‐PTUKI‐100274896 and P‐PTC‐100274048.

## Conflicts of Interest

The authors declare no conflicts of interest.

## Supporting information


**Figure S1:** 2D projection of the embeddings used in the final training dataset and a subset of the embeddings of the entire dataset (one 3‐s audio segment per file) using t‐SNE (Perplexity = 30).
**Figure S2:** Nightly *Pseudophryne covacevichae* detections (green) alongside scaled daily SILO rainfall (blue) and SILO daily minimum temperature (orange). Rainfall and minimum temperature were linearly rescaled to match the range of nightly detections for visual comparison.
**Figure S3:** Complete recording schedules for each automated recording unit. Blue horizontal bars show the full date range of recordings for each site in the Atherton Tableland and orange show the Paluma Range sites. Dashed vertical lines indicate the study period (06/11/2022–06/11/2023), which was selected to maximise data completeness while retaining a full 12 months of recordings where possible.
**Figure S4:** Results from climwin analyses testing rainfall (25–0 days), minimum temperature (29–0 days) and vapour pressure deficit (30–13 days) as predictors of *Pseudophryne covacevichae* calling intensity at sites in the Atherton Tablelands. All predictors had a positive effect on calling intensity. Heatmaps show the relative support (ΔAICc) for climatic windows opening (*y*‐axis) and closing (*x*‐axis) in days before each survey. Warmer colours indicate stronger support compared to the null model. The histogram below each respective heatmap show the distribution of ΔAICc from 100 randomised datasets compared to the observed model (dotted line).
**Figure S5:** Results from climwin analyses testing rainfall (30–11 days), minimum temperature (8–1 days) and vapour pressure deficit (3–0 days) as predictors of *Pseudophryne covacevichae* calling intensity at sites in the Paluma Range. All predictors had a negative effect on calling intensity. Heatmaps show the relative support (ΔAICc) for climatic windows opening (*y*‐axis) and closing (*x*‐axis) in days before each survey. Warmer colours indicate stronger support compared to the null model. The histogram below each respective heatmap show the distribution of ΔAICc from 100 randomised datasets compared to the observed model (dotted line).
**Figure S6:** Example of two *Pseudophryne covacevichae* automated recording unit (ARU) sites, both photos are taken on the 8th August 2023. Photograph (a) is site AT001 and represents a mesic site; Photograph (b) is site AT002 and represents a xeric site.
**Table S1:** Subset of randomisation (randwin) results from the climwin analysis. The first five of 100 randomisations are shown for each region and climate variable. In all cases, improvements in model fit (ΔAICc) were substantially smaller than those obtained in the original sliding window analyses, indicating that the observed climate relationships are unlikely to have arisen by chance.
**Table S2:** Effects of short‐term environmental variables on the calling intensity of *Pseudophryne covacevichae* in the Atherton Tableland and Paluma Range. Paluma Range also has seasonal effects (sin and cos DOY). Shown as standardised coefficients with standard error (*β* ± SE) and test statistics (*Z*, *p*).
**Table S3:** Regional ecosystem descriptions for each automated recording unit used in the study of *P. covacevichae* phenology. Descriptions are taken from the Queensland Government portal: https://apps.des.qld.gov.au/regional‐ecosystems/.
**Table S4:** Bureau of Meteorology climate statistics for average monthly rainfall (mm) at weather stations within the *Pseudophryne covacevichae* study area. The station name and number are listed beside the town name with the average monthly and annual rainfall reported. Data obtained from monthly estimates using https://www.bom.gov.au/climate/data/ portal.
**Table S5:** Monthly rainfall totals from SILO data at each study site, ordered by highest to lowest rainfall (mm) per region. Bureau of Meteorology rainfall totals are included (highlighted) from nearby weather stations. The station name and number are listed beside the town name. Bureau of Meteorology data was obtained from https://www.bom.gov.au/climate/data/ portal. Cells with NA indicate where no vocalisations were detected, due to recorder failure, hence no climate data were used.

## Data Availability

We provide all acoustic recogniser models, training data, example R scripts, and user instructions under open access here: https://zenodo.org/records/19363487. The repository also contains the data and code used in our analyses.
