# Supplementary material for: Automated Acoustic Recogniser Reveals Spatial Variation in Seasonal Calling of the Range‐Restricted Magnificent Broodfrog ( Pseudophryne covacevichae )
Source: Ecol Evol. 2026 Aug 16;16(8):e74191. doi: 10.1002/ece3.74191 (PMC13477864; doi:10.1002/ece3.74191)
Supplement: Supplementary file 1 [file ECE3-16-e74191-s001.docx]

**Online supplementary material for**: Automated acoustic recogniser reveals spatial variation in seasonal calling of the range-restricted magnificent broodfrog (*Pseudophryne covacevichae*)

**Emily R Rush**^1^**, Will Edwards**^1^**, Lin Schwarzkopf**^2^ **& Slade Allen-Ankins**^2^

^1^College of Science & Engineering, James Cook University, Cairns, Qld 4870, Australia

^2^College of Science & Engineering, James Cook University, Townsville, Qld 4811, Australia

**Figure S1.** 2D projection of the embeddings used in the final training dataset and a subset of the embeddings of the entire dataset (one 3-second audio segment per file) using t-SNE (Perplexity = 30).

**Figure S2.** Nightly *Pseudophryne covacevichae* detections (green) alongside scaled daily SILO rainfall (blue) and SILO daily minimum temperature (orange). Rainfall and minimum temperature were linearly rescaled to match the range of nightly detections for visual comparison.

**Figure S3.** Complete recording schedules for each automated recording unit. Blue horizontal bars show the full date range of recordings for each site in the Atherton Tableland and orange show the Paluma Range sites. Dashed vertical lines indicate the study period (06/11/2022 – 06/11/2023), which was selected to maximise data completeness while retaining a full 12 months of recordings where possible.

**Figure S4.** Results from climwin analyses testing rainfall (25-0 days), minimum temperature (29-0 days), and vapour pressure deficit (30-13 days) as predictors of *Pseudophryne covacevichae* calling intensity at sites in the Atherton Tablelands. All predictors had a positive effect on calling intensity. Heatmaps show the relative support (ΔAICc) for climatic windows opening (y-axis) and closing (x-axis) in days before each survey. Warmer colours indicate stronger support compared to the null model. The histogram below each respective heatmap show the distribution of ΔAICc from 100 randomised datasets compared to the observed model (dotted line).

**Figure S5.** Results from climwin analyses testing rainfall (30-11 days), minimum temperature (8-1 days), and vapour pressure deficit (3-0 days) as predictors of *Pseudophryne covacevichae* calling intensity at sites in the Paluma Range. All predictors had a negative effect on calling intensity. Heatmaps show the relative support (ΔAICc) for climatic windows opening (y-axis) and closing (x-axis) in days before each survey. Warmer colours indicate stronger support compared to the null model. The histogram below each respective heatmap show the distribution of ΔAICc from 100 randomised datasets compared to the observed model (dotted line).

**Figure S6.** Example of two *Pseudophryne covacevichae* automated recording unit (ARU) sites, both photos are taken on the 8^th^ August 2023. Photograph a) is site AT001 and represents a mesic site; Photograph b) is site AT002 and represents a xeric site.

**Table S1.** Subset of randomisation (randwin) results from the climwin analysis. The first five of 100 randomisations are shown for each region and climate variable. In all cases, improvements in model fit (ΔAICc) were substantially smaller than those obtained in the original sliding window analyses, indicating that the observed climate relationships are unlikely to have arisen by chance.

| **Region and climate variable** | **∆AICc** | **WindowOpen** | **WindowClose** | **ModelBeta** | **Std.Error** | **ModelInt** | **ModWeight** | **Repeat** |
| --- | --- | --- | --- | --- | --- | --- | --- | --- |
| Atherton Tablelands Rainfall | -2.458132634 | 28 | 26 | -0.3194636 | 0.143692717 | 3.681072186 | 0.015528665 | 1 |
|  | -0.678398436 | 12 | 12 | 0.163361885 | 0.105690192 | 3.68904409 | 0.006215338 | 2 |
|  | -1.387539696 | 18 | 17 | 0.207439785 | 0.119297282 | 3.687774662 | 0.007803846 | 3 |
|  | -3.310174561 | 22 | 20 | 0.310036409 | 0.142206601 | 3.68326812 | 0.011874179 | 4 |
|  | 0.075725106 | 14 | 14 | 0.131161442 | 0.09968456 | 3.68987912 | 0.004307272 | 5 |
| Atherton Tablelands minTemp | 0.225258785 | 23 | 23 | -0.094856063 | 0.071976801 | 3.685214392 | 0.004097163 | 1 |
|  | 0.821373848 | 1 | 1 | 0.077001299 | 0.070207775 | 3.700459493 | 0.003262364 | 2 |
|  | 1.466755676 | 18 | 18 | -0.050342412 | 0.06893266 | 3.688618723 | 0.002596916 | 3 |
|  | -0.900206479 | 7 | 7 | 0.125478546 | 0.07278143 | 3.698649747 | 0.006496042 | 4 |
|  | 1.273334641 | 17 | 17 | -0.05658901 | 0.066607546 | 3.68788791 | 0.002824299 | 5 |
| Atherton Tablelands VPD | 0.209031679 | 30 | 30 | 0.122347562 | 0.092374801 | 3.720283642 | 0.00457372 | 1 |
|  | 1.178513687 | 12 | 12 | -0.078031377 | 0.085033202 | 3.670787879 | 0.002968618 | 2 |
|  | 0.40558613 | 27 | 25 | -0.125538921 | 0.098214995 | 3.656186312 | 0.003989484 | 3 |
|  | 0.728359482 | 8 | 8 | -0.095836339 | 0.083903359 | 3.666205453 | 0.00347072 | 4 |
|  | -1.324170412 | 28 | 25 | -0.190719941 | 0.103005813 | 3.635958778 | 0.007601046 | 5 |
| Paluma Range Rainfall | -19.70689943 | 8 | 8 | -0.159678941 | 0.030785815 | 5.176541274 | 0.768563911 | 1 |
|  | -17.63279663 | 6 | 6 | 0.117748686 | 0.029521278 | 5.182539153 | 0.319683692 | 2 |
|  | -12.56166983 | 18 | 18 | -0.121370652 | 0.029308761 | 5.183543744 | 0.62018373 | 3 |
|  | -5.750702562 | 10 | 9 | -0.111978352 | 0.038495455 | 5.184557617 | 0.042666433 | 4 |
|  | -7.749551995 | 22 | 21 | 0.113836669 | 0.03810027 | 5.183906185 | 0.048692139 | 5 |
| Paluma Range minTemp | -1.655335077 | 12 | 12 | 0.057767712 | 0.029992313 | 5.194927013 | 0.009837053 | 1 |
|  | 0.578041762 | 24 | 24 | 0.034331697 | 0.028533786 | 5.192136922 | 0.003664788 | 2 |
|  | -1.825532243 | 5 | 5 | 0.059600207 | 0.030265389 | 5.196110276 | 0.010008977 | 3 |
|  | -3.020163569 | 17 | 17 | 0.063899592 | 0.028225395 | 5.194402303 | 0.010566878 | 4 |
|  | -2.640527009 | 19 | 19 | -0.059165053 | 0.027674949 | 5.180337672 | 0.012741526 | 5 |
| Paluma Range VPD | -16.01013436 | 18 | 18 | -0.13881477 | 0.032136654 | 5.163878431 | 0.692573473 | 1 |
|  | -6.435421248 | 1 | 0 | 0.100746083 | 0.034999026 | 5.195842551 | 0.018723527 | 2 |
|  | -13.37308182 | 21 | 18 | 0.149476995 | 0.038668325 | 5.200908206 | 0.164394153 | 3 |
|  | -14.51095479 | 14 | 14 | 0.118734812 | 0.029887043 | 5.19455379 | 0.759638707 | 4 |
|  | -4.634791131 | 27 | 27 | 0.082562009 | 0.032389797 | 5.198679541 | 0.03327941 | 5 |

**Table S2.** Effects of short-term environmental variables on the calling intensity of *Pseudophryne covacevichae* in the Atherton Tableland and Paluma Range. Paluma Range also has seasonal effects (sin and cos DOY). Shown as standardised coefficients with standard error (β ± SE) and test statistics (Z, p).

| **Predictor** | **β (SE)** | **Z** | ***p*** |
| --- | --- | --- | --- |
| **Atherton Tableland (*n* = 7)** | | | |
| Intercept | 3.134 (0.473) | 6.62 | < 0.001 |
| Rainfall | 2.357 (0.146) | 16.10 | < 0.001 |
| minTemp | 0.602 (0.090) | 6.63 | < 0.001 |
| Vapour pressure deficit | 0.822 (0.068) | 11.92 | < 0.001 |
| Recorder | 0.474 (0.716) | 0.66 | 0.507 |
| **Paluma Range (*n* = 3)** | | | |
| Intercept | 4.621 (0.117) | 39.47 | < 0.001 |
| Rainfall | -0.857 (0.131) | -6.56 | < 0.001 |
| minTemp | -0.184 (0.071) | -2.61 | 0.009 |
| Vapour pressure deficit | -0.656 (0.064) | -10.9 | < 0.001 |
| sinDOY | 0.562 (0.068) | 8.29 | < 0.001 |
| cosDOY | -0.372 (0.121) | -3.07 | 0.002 |

**Table S3.** Regional ecosystem descriptions for each automated recording unit used in the study of *P. covacevichae* phenology. Descriptions are taken from the Queensland Government portal: https://apps.des.qld.gov.au/regional-ecosystems/

| **Site** | **RE code** | **Short description** | **Description** | **Biodiversity status** |
| --- | --- | --- | --- | --- |
| AT001  (Mesic) | 7.12.52 | Eucalyptus resinifera, Corymbia intermedia, Allocasuarina littoralis, Syncarpia glomulifera, E. drepanophylla +/- E. reducta woodland on granite and rhyolite in the dry to moist rainfall zone | Eucalyptus resinifera (red mahogany), Corymbia intermedia (pink bloodwood), Allocasuarina littoralis, Syncarpia glomulifera (turpentine), E. drepanophylla (ironbark) +/- E. reducta (Queensland stringybark) woodland. Hills on granite and rhyolite, in the dry to moist rainfall zone. Not a Wetland. (BVG1M: 8a). | Of concern |
| AT002  (Xeric) | 7.12.65a | Rock pavement or areas of skeletal soil on granite and rhyolite of dry western or southern areas +/- shrublands to closed forests of Acacia spp. And/or Lophostemon suaveolens and/or Allocasuarina littoralis and/or Eucalyptus lockyeri subsp. Exuta | Rock pavement communities of the dry rainfall zone with Acacia leptostachya, Eucalyptus lockyeri subsp. exuta, Lophostemon confertus, L. suaveolens, Persoonia falcata, Ficus rubiginosa and Allocasuarina inophloia. Occurs on rock pavements of rhyolite ranges. Not a Wetland. (BVG1M: 29b). | Of concern |
| AT003  (Xeric) | 7.12.30a | Corymbia citriodora +/- Eucalyptus portuensis woodland to open forest on granite and rhyolite | Corymbia citriodora, Eucalyptus portuensis, C. intermedia, Syncarpia glomulifera woodland to low woodland to open forest with Callitris intratropica, Acacia calyculata and Xanthorrhoea johnsonii. Uplands and highlands, of the moist and dry rainfall zones. Not a Wetland. (BVG1M: 10b). | No concern at present |
| AT004  (Mesic) | 7.12.58 | Eucalyptus reducta +/- E. granitica +/- Corymbia dimorpha +/- C. citriodora woodland to open forest on granite and rhyolite | Eucalyptus reducta woodland to open forest (6-18m tall). Common associated species include E. granitica, Corymbia dimorpha, C. citriodora, E. cloeziana and occasionally C. intermedia. There is often a sparse secondary tree layer of C. abergiana and/or E. lockyeri. There may be a very sparse tall shrub layer of species such as Acacia flavescens, Persoonia falcata, Allocasuarina littoralis and Acacia simsii, and a very sparse to dense lower shrub layer of Acacia calyculata, Pultenaea millarii, Jacksonia thesioides, Grevillea glossadenia, Grevillea dryandri subsp. dryandri, Homoranthus porteri and Dodonaea lanceolata var. subsessilifolia. The ground layer is often dominated by species such as Themeda triandra, Eriachne spp., Cleistochloa subjuncea, Lomandra longifolia, Mnesithea rottboellioides, Xanthorrhoea johnsonii, Heteropogon triticeus and Coronidium newcastlianum. Granite and rhyolite. Not a Wetland. (BVG1M: 9d). | Of concern |
| AT005  (Xeric) | 9.12.30a  9.12.20  9.12.30b | **9.12.30a & b**: Mixed woodland to open forest of Corymbia leichhardtii (yellowjacket) and Eucalyptus cloeziana (Gympie messmate) +/- Eucalyptus spp. and Corymbia spp. as subdominants. The shrub layer is sparse to mid-dense with a shrubland of Acacia spp. sometimes occurring. The ground layer is grassy. Occurs on rocky hills to steep hills on igneous geologies. **9.12.20:** Eucalyptus pachycalyx +/- E. cloeziana +/- Corymbia leichhardtii woodland on steep igneous hills | **9.12.30a:** Woodland to open forest of Corymbia leichhardtii (yellowjacket) and Eucalyptus cloeziana (Gympie messmate) +/- E. portuensis (white mahogany) +/- C. citriodora subsp. citriodora (lemon-scented gum) +/- E. cullenii (Cullen's ironbark) +/- Callitris intratropica (cypress pine). Some canopy species can occur as emergents. The sparse to mid-dense shrub layer is dominated by juvenile canopy species, Persoonia falcata, Grevillea glauca (bushman's clothes peg) and Allocasuarina inophloia (stringybark sheoak) and a lower shrub with Jacksonia thesioides and Xanthorrhoea johnsonii (grass-tree) can occur. The sparse to mid-dense ground layer is dominated by Themeda triandra (kangaroo grass). Rocky rhyolite hills to steep hills. Not a Wetland. (BVG1M: 13a). **9.12.30b:** Shrubland of Acacia leptostachya (slender wattle) +/- A. umbellata +/- Callitris intratropica (cypress pine) emergents. There is no mid layer or ground layer. Occurs on shallow soils on rock pavements within 9.12.30a. Not a Wetland. (BVG1M: 24a). **19.12.20:** Woodland to low woodland of Eucalyptus pachycalyx (pumpkin gum) +/- E. cloeziana (Gympie messmate) +/- Corymbia leichhardtii (yellowjacket) +/- Callitris intratropica (cypress pine) +/- E. portuensis (white mahogany) +/- E. cullenii (Cullen's ironbark) or E. atrata. The mid-dense shrub layer includes juvenile canopy species, Grevillea glauca (bushman's clothes peg), Persoonia falcata and Xanthorrhoea johnsonii (grass-tree). The medium to dense grassy ground layer is mostly dominated by Themeda triandra (kangaroo grass). Occurs on steep rugged hills on igneous hills. Not a Wetland. (BVG1M: 13a). | No concern at present |
| AT006  (Xeric) | 7.12.30a | Corymbia citriodora +/- Eucalyptus portuensis woodland to open forest on granite and rhyolite | Corymbia citriodora, Eucalyptus portuensis, C. intermedia, Syncarpia glomulifera woodland to low woodland to open forest with Callitris intratropica, Acacia calyculata and Xanthorrhoea johnsonii. Uplands and highlands, of the moist and dry rainfall zones. Not a Wetland. | No concern at present |
| AT007  (Mesic) | 7.12.52 | Eucalyptus resinifera, Corymbia intermedia, Allocasuarina littoralis, Syncarpia glomulifera, E. drepanophylla +/- E. reducta woodland on granite and rhyolite in the dry to moist rainfall zone | Eucalyptus resinifera (red mahogany), Corymbia intermedia (pink bloodwood), Allocasuarina littoralis, Syncarpia glomulifera (turpentine), E. drepanophylla (ironbark) +/- E. reducta (Queensland stringybark) woodland. Hills on granite and rhyolite, in the dry to moist rainfall zone. Not a Wetland. (BVG1M: 8a). | Of concern |
| PR001  (Mesic) | 7.12.27a | Eucalyptus reducta open forest to woodland on uplands and highlands on shallow granitic and rhyolitic soils | Eucalyptus reducta medium open forest and woodland. Uplands and highlands on shallow granitic and rhyolitic soils, of the moist rainfall zone. Not a Wetland. (BVG1M: 9d). | No concern at present |
| PR002  (Mesic) | 7.12.51b | Eucalyptus resinifera, Syncarpia glomulifera, E. portuensis, Corymbia abergiana +/- C. leptoloma woodland of rocky hills on granite and rhyolite in the Paluma-Seaview (south-west) subregion | Eucalyptus resinifera, Syncarpia glomulifera, Corymbia leptoloma, C. intermedia, C. abergiana, E. portuensis, medium woodland with Banksia aquilonia, Allocasuarina littoralis, Lophostemon confertus and Xanthorrhoea johnsonii. Rocky hillslopes of the dry rainfall zone. Not a Wetland. (BVG1M: 8a). | Of concern |
| PR003  (Mesic) | 7.12.24a | Eucalyptus portuensis and Corymbia intermedia open forest to woodland (or vine forest with E. portuensis and C. intermedia emergents) on foothills and uplands on granites and rhyolites | Eucalyptus portuensis, Corymbia intermedia, E. drepanophylla, E. platyphylla, E. tereticornis, C. tessellaris, Lophostemon suaveolens, Syncarpia glomulifera open forest to woodland. Foothills, of the wet and moist rainfall zones. Not a Wetland. (BVG1M: 9d) | No concern at present |

**Table S4.** Bureau of Meteorology climate statistics for average monthly rainfall (mm) at weather stations within the *Pseudophryne covacevichae* study area. The station name and number are listed beside the town name with the average monthly and annual rainfall reported. Data obtained from monthly estimates using <https://www.bom.gov.au/climate/data/> portal.

| **Town, station & statistic** | **Jan** | **Feb** | **Mar** | **Apr** | **May** | **Jun** | **Jul** | **Aug** | **Sep** | **Oct** | **Nov** | **Dec** | **Annual (mm)** |
| --- | --- | --- | --- | --- | --- | --- | --- | --- | --- | --- | --- | --- | --- |
| **Herberton (Alert) 31198** | | | | | | | | | | | | | |
| Mean rainfall (mm) | 200.3 | 202.1 | 132.9 | 53 | 22.2 | 15.5 | 14.4 | 7.5 | 7.6 | 26.4 | 48.4 | 147.1 | 859.1 |
| **Ravenshoe (Alert) 31200** | | | | | | | | | | | | | |
| Mean rainfall (mm) | 276.8 | 310.3 | 274.7 | 125 | 74.1 | 56 | 51.4 | 32.7 | 31.7 | 46.2 | 59.6 | 175.4 | 1551.6 |
| **Paluma (Ivy Cottage) 32064** | | | | | | | | | | | | | |
| Mean rainfall (mm) | 570.4 | 642.1 | 417.1 | 173.4 | 101.5 | 64.3 | 63.2 | 46.4 | 57.6 | 85.8 | 164.6 | 296.8 | 2711 |

**Table S5.** Monthly rainfall totals from SILO data at each study site, ordered by highest to lowest rainfall (mm) per region. Bureau of Meteorology rainfall totals are included (highlighted) from nearby weather stations. The station name and number are listed beside the town name. Bureau of Meteorology data was obtained from <https://www.bom.gov.au/climate/data/> portal. Cells with NA indicate where no vocalisations were detected, due to recorder failure, hence no climate data were used.

| **Site/Town** | **Nov 2022** | **Dec 2022** | **Jan 2023** | **Feb 2023** | **Mar 2023** | **Apr 2023** | **May 2023** | **Jun 2023** | **Jul 2023** | **Aug 2023** | **Sep 2023** | **Oct 2023** | **Nov 2023** | **Total (mm)** |
| --- | --- | --- | --- | --- | --- | --- | --- | --- | --- | --- | --- | --- | --- | --- |
| **Herberton (Alert) 31198** | 85 | 54 | 400 | 298 | 107 | 140 | 7 | 7 | 37 | 30 | 15 | 2 | 52 | 1234 |
| **Ravenshoe (Alert) 31200** | 82 | 185 | 404 | 496 | 180 | 165 | 47 | 51 | 158 | 106 | 71 | 18 | 72 | 2035 |
| AT004 | 92.8 | 196.7 | 402.6 | 453.6 | 151.2 | 154.3 | 34 | 31.9 | 114.4 | 76.7 | 52 | 10.3 | 0 | 1770.5 |
| AT005 | 106.1 | 185.6 | 408.1 | 377 | 155.9 | 146.8 | 23.2 | 23.9 | 77.1 | 73 | 42.8 | 7.8 | 0.1 | 1627.4 |
| AT002 | 105.5 | 187.1 | 454.6 | 337.8 | 150.5 | 145.2 | 17 | 17.2 | 59.8 | 65.2 | 36.5 | 6.2 | NA | 1582.6 |
| AT006 | 104.4 | 192.3 | 427 | 343.2 | 135.8 | 141.9 | 22.8 | 19.1 | 71.3 | 54.8 | 34 | 5.6 | 0 | 1552.2 |
| AT007 | 114.8 | 189.4 | 425.3 | 335.4 | 121.3 | 131.8 | 22.6 | 16.9 | 78.7 | 56 | 29 | 4.2 | 0 | 1525.4 |
| AT001 | 102.9 | 195.8 | 434.3 | 307.9 | 146.9 | 158.9 | 17.6 | 10.2 | 61.1 | 42.7 | 1 | NA | NA | 1479.3 |
| AT003 | NA | NA | NA | 149 | 155.9 | 146.8 | 23.2 | 23.9 | 77.1 | 73 | 10.3 | NA | NA | 659.2 |
| **Paluma (Ivy Cottage) 32064** | 146.4 | 297.2 | 597.2 | 571.6 | 382.4 | 61 | 24 | 127.4 | 162.2 | 22.4 | 40.2 | 70.6 | 140.2 | 2642.8 |
| PR002 | 112.6 | 139.6 | 348.3 | 340 | 160.5 | 26.1 | 16.5 | 35.5 | 104.5 | 13.9 | 9.7 | 6.9 | 1 | 1315.1 |
| PR001 | 88.7 | 108.7 | 323.5 | 283.4 | 109.5 | 20.2 | 6.5 | 18.4 | 84 | 9.2 | 6.5 | 4.3 | 0.8 | 1063.7 |
| PR003 | 88.7 | 108.7 | 323.5 | 283.4 | 109.5 | 20.2 | 6.5 | 18.4 | 84 | 9.2 | 6.5 | 4.3 | 0.8 | 1063.7 |
